# Plant Silicon Defences Suppress Herbivore Growth but Trigger Compensatory Feeding in a Moderate-Accumulating Grass

**DOI:** 10.3390/plants15091380

**Published:** 2026-04-30

**Authors:** Scott N. Johnson, Ximena Cibils-Stewart, Jannatul Ferdous

**Affiliations:** 1 Hawkesbury Institute for the Environment, Western Sydney University, Penrith, NSW 2751, Australia; xcibils@inia.org.uy (X.C.-S.); j.ferdous@westernsydney.edu.au (J.F.); 2 Instituto Nacional de Investigación Agropecuaria (INIA), La Estanzuela Research Station, Colonia 700000, Uruguay

**Keywords:** herbivore, feeding behaviour, herbivore growth rates, insect herbivore, nutrients, plant defence, silica

## Abstract

Silicon (Si) accumulation is a widespread anti-herbivore defence in grasses, yet little is known about how insects counteract silicification, including via compensatory feeding, or whether Si-mediated changes in plant stoichiometry also influence herbivore performance. We examined how Si supplementation alters foliar Si, carbon (C), nitrogen (N), and phosphorus (P) in two grasses with contrasting accumulation strategies, *Brachypodium distachyon* (high accumulator) and *Lolium arundinaceum* (moderate accumulator), and the consequences for growth and feeding by *Helicoverpa armigera*. Plants were grown hydroponically with or without Si, and herbivore relative growth rate (RGR), relative consumption (RC), and Efficiency of Conversion of Ingested food (ECI) were measured. Si supplementation had stronger effects on herbivore performance in *B. distachyon* compared with *L. arundinaceum*. RGR declined by 126% on *B. distachyon* compared with 40% on *L. arundinaceum*. Herbivores increased RC on Si-supplemented *L. arundinaceum*, with RC positively correlated with foliar Si concentrations, but no compensatory feeding occurred on *B. distachyon*. N and P concentrations were positively correlated with RGR in *L. arundinaceum* and ECI in *B. distachyon*. In conclusion, the degree of Si accumulation in grasses influences both plant stoichiometry and has contrasting impacts on herbivore feeding strategies.

## 1. Introduction

Plants deploy an arsenal of physical and chemical defences to resist attack by herbivorous animals, sometimes referred to as the reason for ‘why the world is green’ [1]. Understanding these anti-herbivore defences, and how herbivores attempt to counteract them, is important in many disciplines ranging from crop protection, where losses to arthropod pests can exceed 15% annually [2], to evolutionary biology. The grasses (Poaceae) are a crucial group of plants in both natural and managed systems, including many pasture species and cereal crops. For example, just three major cereal crops provide 42% of human calories [3]. Many Poaceae are primarily reliant on silicon (Si) anti-herbivore defences [4,5], which they accumulate from the soil and deposit in various plant tissues [6].

Silicified plant tissues are abrasive to arthropod herbivores and can wear down mouthparts, inhibit feeding, and reduce nutrient acquisition once ingested [7,8]. Moreover, Si accumulation may enhance the functionality of secondary metabolite defensive pathways in the plant to augment other types of defence [9,10], even including indirect defences that involve recruitment of pests’ natural enemies [11,12]. Si accumulation can also cause changes in tissue stoichiometry, particularly the balance between carbon (C), nitrogen (N), and phosphorus (P) [6]. In general, Si accumulation leads to declines in C content [13], whereas N and P concentrations have been reported to increase, decrease, or remain unchanged with Si supplementation [14,15,16,17]. Given that N is often a limiting nutrient in insect herbivore diets [18] and P is increasingly recognised as an important dietary component [19], Si-driven changes in these nutrients may therefore also influence insect herbivore performance.

While many studies report negative impacts of Si accumulation in plants on insect herbivore performance across many taxa, most address a limited repertoire of performance traits. A meta-analysis [20] exploring the impacts of Si defences on herbivores indicated that feeding behaviour was not commonly measured, with notable exceptions [8]. In particular, Relative Consumption (RC) of plant tissues and the Efficiency of Conversion of Ingested food (ECI), the proportion of consumed food converted into body biomass [21], represented just 3% and 2% of measured responses, respectively [20]. Quantifying these feeding behaviours could be important, however, because many insect herbivores engage in compensatory feeding (e.g., increased RC) when challenged by inferior quality host plants [22]. This is consequential because increased feeding rates on Si-supplemented plants may produce a net negative impact for plant fitness, even when insect herbivore performance (e.g., Relative Growth Rates; RGR) is negatively affected. Conversely, herbivore fitness may still be negatively impacted even when undertaking compensatory feeding if ECI remains low.

This study aimed to address these knowledge gaps by investigating how Si accumulation in a moderate (*Lolium arundinaceum*) [23,24,25] and high Si-accumulating (*Brachypodium distachyon*) [26,27,28,29,30] grass species affected the growth and feeding performance of a generalist insect herbivore, the cotton bollworm (*Helicoverpa armigera*). We hypothesise that *H. armigera* RGR and ECI will be suppressed by Si supplementation on both species, with sharper declines on the high Si-accumulating grass (*B. distachyon*). We further hypothesise that RC increases when feeding on Si-supplemented *L. arundinaceum*, reflecting a compensatory feeding strategy, but this is not possible on *B. distachyon* due to higher levels of silicification. Depending on how Si supplementation affects N and P concentrations, this may have additional impacts on herbivore performance.

## 2. Results

### 2.1. Plant Elemental Chemistry

**Figure 1:**
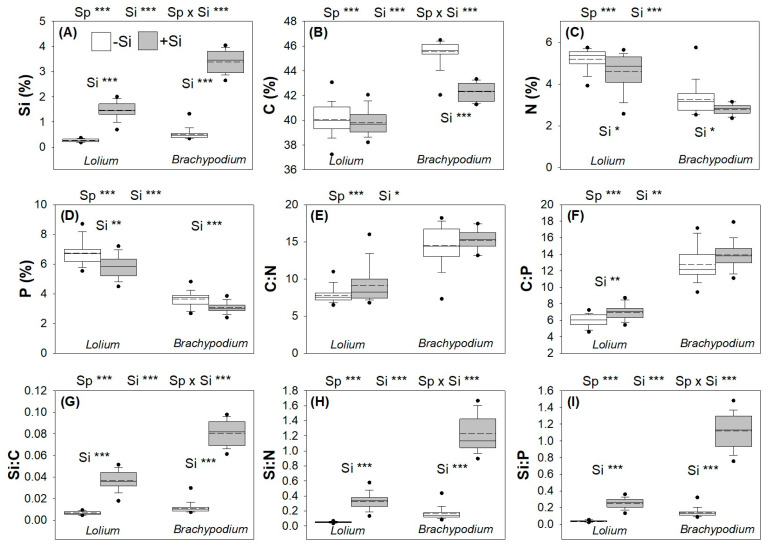
Impacts of Si supplementation (shaded bars) on leaf chemistry of *L. arundinaceum* (labelled *Lolium*) and *B. distachyon* (labelled *Brachypodium*). Dashed lines represent mean values; solid lines depict the inclusive median. Leaf concentrations (% dry mass) of (**A**) Si, (**B**) C, (**C**) N, and (**D**) P are shown, followed by elemental ratios (**E**) C:N, (**F**) C:P, (**G**) Si:C, (**H**) Si:N and (**I**) Si:P. Sample size: N = 17. Statistically significant differences (see Table S1) between plant species (Sp) and Si supplementation (Si) are indicated above each panel for the two species collectively, and within panels for each species separately (Si only). *** *p* < 0.001, ** *p* < 0.01 and * *p* < 0.05.

There were substantial differences in elemental chemistry between the two plant species (Figure 1; Table S1), with Si supplementation also affecting all of the elements we quantified (Table S1). Expectedly, Si supplementation increased Si concentrations in both plants, but the increase was substantially higher in *B. distachyon* (Figure 1A; Table S1). Overall, Si supplementation suppressed leaf C concentrations (Table S1), but this was largely due to the 7.1% decrease in *B. distachyon* compared to the negligible decrease (0.6%) observed in *L. arundinaceum* (Figure 1B). Leaf N concentrations decreased with Si supplementation in both species (Figure 1C; Table S1). P concentrations were suppressed by Si supplementation to a similar extent in both species (Figure 1D). Si supplementation slightly increased the C:N ratio in *L. arundinaceum* but not in *B. distachyon* (Figure 1E), mostly due to the larger impacts of Si supplementation on C concentrations in *B. distachyon*. The C:P ratio rose with Si supplementation in *L. arundinaceum* (Figure 1F). The ratio of C, N, and P to Si concentrations (Figure 1G, Figure 1H, and Figure 1I, respectively) was substantially higher in *B. distachyon* due to the greater increase in Si concentrations, reflected in the statistically significant interaction between plant species and Si supplementation.

### 2.2. Herbivore Growth and Feeding Performance

**Figure 2:**
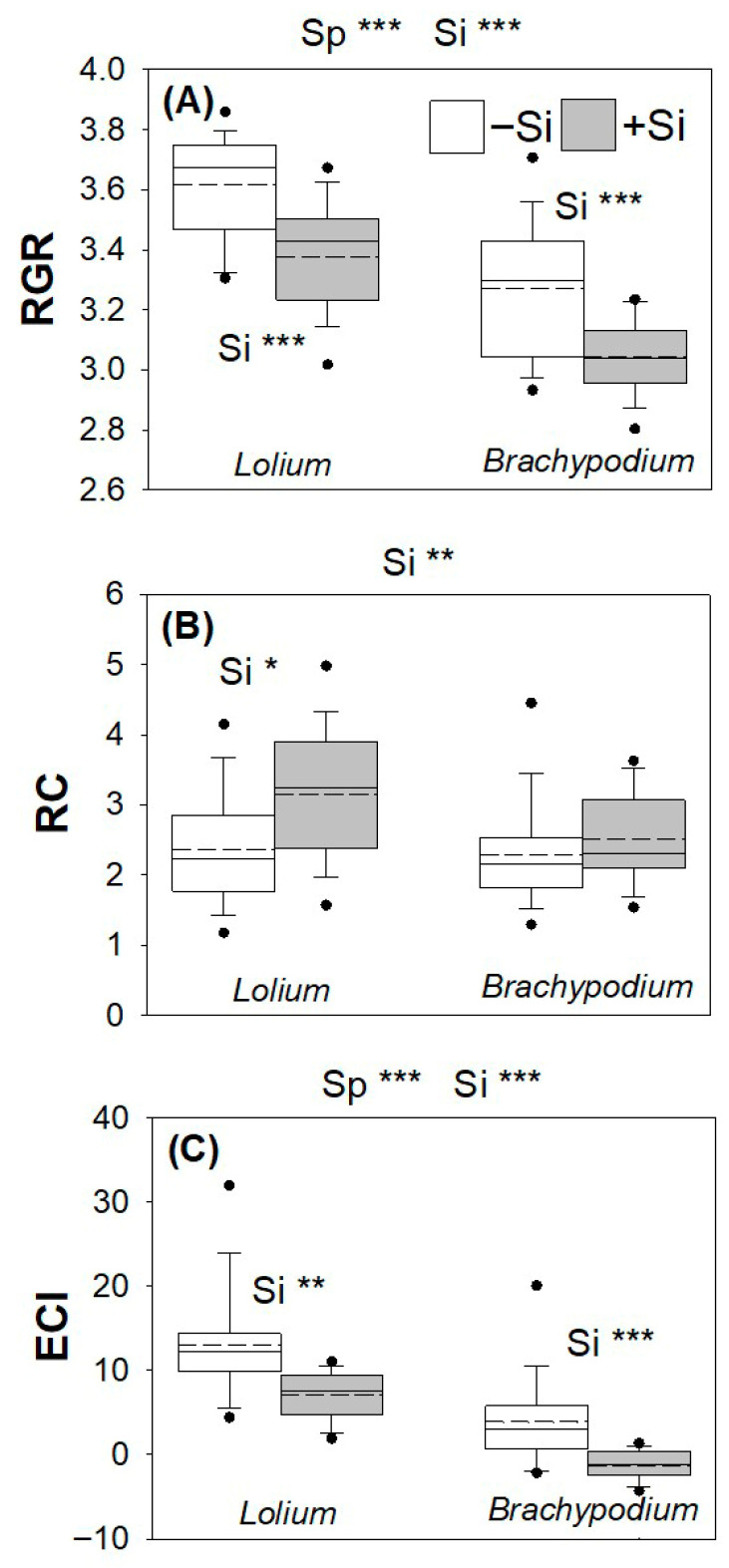
Impacts of Si supplementation (shaded bars) on the performance of *Helicoverpa armigera* feeding on *L. arundinaceum* and *B. distachyon*, depicting (**A**) relative growth rates; RGR (mg^−1^ mg^−1^ day^−1^), (**B**) relative consumption; RC, and (**C**) efficiency of conversion of ingested food; ECI. Dashed lines represent mean values; solid lines depict the inclusive median. Sample size: N = 16–17. Statistically significant differences (see Table S1) between plant species (Sp) and Si supplementation (Si) are indicated above each panel for the two species collectively, and within panels for each species separately (Si only). *** *p* < 0.001, ** *p* < 0.01 and * *p* < 0.05.

Relative growth rates (RGR) were lower in *B. distachyon* compared to *L. arundinaceum* overall (Figure 2A; Table S1). Si supplementation reduced RGR by 126% in *B. distachyon* and by 40% in *L. arundinaceum* (Figure 2A; Table S1). Relative consumption (RC) increased when *H. armigera* were feeding on Si-supplemented *L. arundinaceum* plants but not when feeding on *B. distachyon* (Figure 2B; Table S1). Si supplementation impaired the insects’ ability to convert food into body mass, reflected in a 65% decrease in ECI across both plant species, but ECI was already 45% lower in *B. distachyon* compared to *L. arundinaceum* even without Si supplementation (Figure 2C; Table S1).

### 2.3. Correlations Between Plant Chemistry and Herbivore Responses

**Figure 3:**
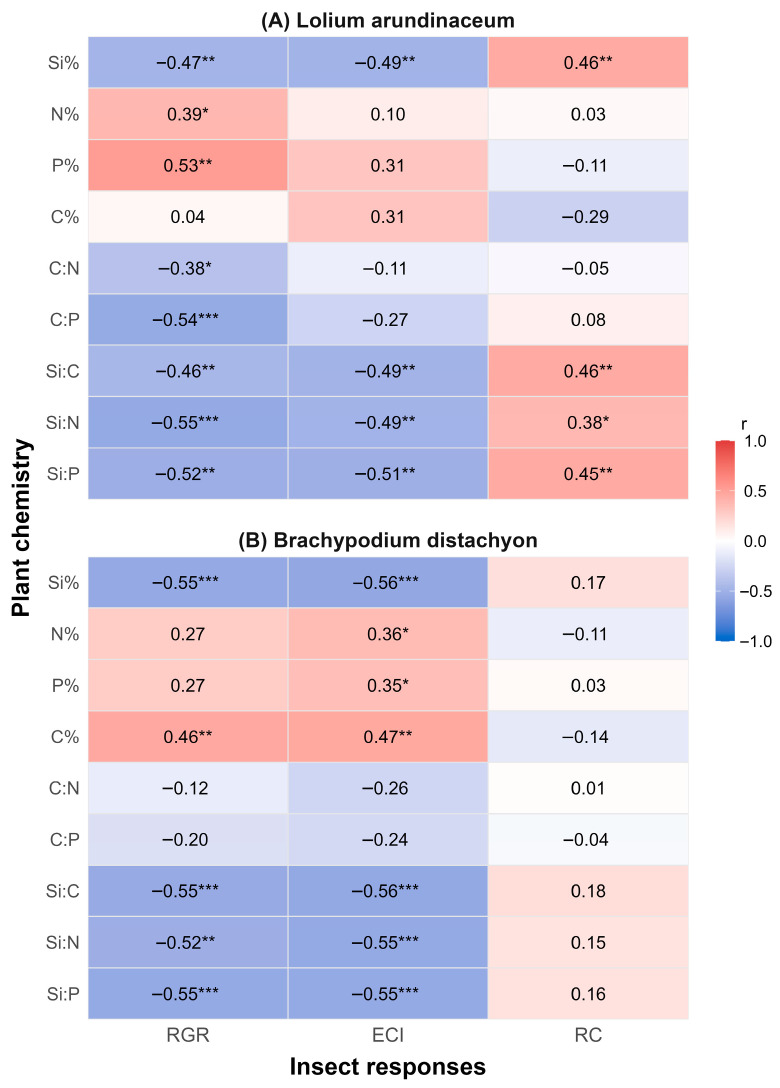
Pearson correlation coefficients (*r*) between the three growth metrics (RGR, ECI, and RC; rows) and tissue elemental composition (columns: %Si, %N, %P, %C, C:N, C:P, Si:C, Si:N, and Si:P) for (**A**) *L. arundinaceum* and (**B**) *B. distachyon*. Numbers inside tiles represent *r* values; asterisks indicate two-tailed significance (*p* < 0.05 *, *p* < 0.01 **, *p* < 0.001 ***). Colour scale shows correlation strength and direction of the relationship (blue = negative, white = zero, red = positive). Sample size: N = 33–34.

Si supplementation was negatively correlated with RGR and ECI in both plant species (Figure 3) and positively correlated with RC in *L. arundinaceum* (Figure 3A). Foliar N and P concentrations were positively correlated with RGR in *L. arundinaceum* and ECI (Figure 3A) in *B. distachyon* (Figure 3B). Foliar C concentrations were positively correlated with RGR and ECI in *B. distachyon*, most likely reflecting that higher C plants contained less Si (see Figure 2B).

## 3. Discussion

This study provides further evidence that Si is a potent plant defence against insect herbivores [5,20], but extends our understanding by showing that the degree of Si accumulation affects insect herbivore feeding strategies differently. In particular, the comparatively lower levels of Si defence in *L. arundinaceum* meant that herbivores could increase consumption rates, potentially to also offset Si-mediated decreases in N and P. In contrast, the more substantial increases in foliar Si concentrations in *B. distachyon* prevented increases in consumption, even though decreases in N and P could have made such compensatory feeding somewhat beneficial to the herbivore. The positive correlations of foliar N and P with RGR in *L. arundinaceum* and ECI in *B. distachyon* suggest that nutrient availability within the plant still plays a role, even in the presence of Si defences.

### 3.1. Relative Consumption Rates Increased on the Low Si Accumulating Species

Si accumulation in both plant species led to significant reductions in the RGR of *H. armigera* and there was a negative correlation between the two—more Si accumulation led to steeper declines in RGR. The amount of leaf material consumed (RC), however, increased on Si-supplemented *L. arundinaceum*, and RC was positively correlated with foliar Si concentrations. This most likely reflects compensatory feeding, whereby insect herbivores consume more plant biomass to try to acquire adequate nutrition [22,31]. Si-supplemented *L. arundinaceum* had 57% less Si than Si-supplemented *B. distachyon*, so it may have been comparatively easier to consume more plant tissue on the former.

Compensatory feeding on Si-supplemented plants has also been reported for *Schistocerca gregaria* (desert locust) [7], *S. americana* (American Grasshopper) [32], and *Cnaphalocrocis medinalis* (rice leaf folder) [33]. Furthermore, two of these studies reported that *Spodoptera exempta* (African armyworm) [7] and *S. frugiperda* (fall armyworm) [33] did not display compensatory feeding on the Si-supplemented plants. This difference was mostly attributed to the Orthopteran species (*Schistocerca* spp.) being generalist feeders, which have the capacity to increase consumption rates on nutritionally inferior plants, whereas the Lepidopteran *Spodoptera*, as a more specialist feeder, could not [7]. In the current study, we also used a generalist herbivore (*H. armigera*), which, like *S. gregaria*, was capable of compensatory feeding in response to Si supplementation, but crucially not when feeding on the high Si-accumulating species. This suggests that the Si accumulating strategy of the host plant, in addition to the diet breadth of the herbivore, determines if compensatory feeding is possible.

Si supplementation also increased the ratio of C to N (C:N) by 17% in *L. arundinaceum*, which was also seen in Si-supplemented rice (*Oryza sativa*) plants, and one of the main reasons given for increased consumption by rice leaf folder (*C. medinalis*) [33]. Our results are therefore consistent with this finding since Si supplementation did not increase C:N in *B. distachyon* but tended to increase C:N in *L. arundinaceum*. The situation is analogous to the commonly observed increases in plant C:N under elevated atmospheric CO_2_, which meta-analysis suggests increase by an average of 11% across plant species [34]. These changes are associated with an average 17% increase in relative consumption by insect herbivores [35].

### 3.2. Nutritional Components Affected Herbivore Traits Differently Depending on Si Accumulation

The negative relationship between plant Si accumulation and insect herbivore performance is widely reported, with meta-analysis demonstrating that herbivore performance declines on average by 14–45%, depending on feeding guild [20]. Moreover, several studies have reported direct negative correlations between concentrations of Si in the plant and RGR [16,36,37], although to our knowledge this relationship has not previously been reported between Si concentrations and ECI.

Our results suggest that plant N and P had some positive impacts on *H. armigera*, specifically being positively correlated with RGR in *L. arundinaceum* and ECI in *B. distachyon*, but these effects were overwhelmed by the negative impacts of Si accumulation. Elevated N, and to a lesser extent P, generally enhance insect herbivore ECI by increasing the availability of substrates required for growth [21]. N directly supports protein synthesis, while P underpins ATP production and ribosomal RNA content, reducing the metabolic costs of tissue construction in the herbivore [18,19]. These nutrients positively affected RGR when feeding on *L. arundinaceum* and ECI when feeding on *B. distachyon*. This difference probably reflects that *H. armigera* were able to better cope with the lower levels of Si in *L. arundinaceum* compared to *B. distachyon* (e.g., increased RC) and direct nutrient acquisition towards growth (i.e., RGR). Consumption on *B. distachyon* was very low (45% lower than on *L. arundinaceum*, in the absence of Si), meaning that even small gains in herbivore mass would translate into a disproportionate increase in ECI. The positive correlation between C concentrations in *B. distachyon* and *H. armigera* performance most likely reflects that plants with the lowest Si (i.e., no Si supplementation) had the highest C concentrations.

### 3.3. Experimental Considerations

While many aspects of plant Si defences are conferred by physical fortification of tissues (i.e., leaf trichomes and prickle cells) [38], especially in the current experimental system [27,28], Si accumulation may also be linked to the production of secondary metabolite defences [39,40]. While constitutive secondary metabolite defences would have been present in the excised leaf material used in the feeding assays, any induced defences derived from the main plant could not be delivered to the excised leaves. For that matter, Si defences can be rapidly induced following herbivory [41,42,43], so the impacts of Si supplementation reported here may potentially underestimate those seen in intact plants. For example, RC rates were similar on Si-supplemented and non-supplemented *B. distachyon* plants in the current study, which contrasts with the study by Waterman et al. [29] which reported that leaf consumption declined when feeding on Si-supplemented *B. distachyon*. Feeding assays were conducted in situ in Waterman et al. [29] study, which would have allowed Si induction and may partly explain this discrepancy. Alternatively, Waterman et al. [29] calculated consumption rates based on visual estimates of leaf damage, as opposed to RC, so the results are not strictly comparable. RC and ECI calculations are only possible using ex situ assays since the mass of consumed plant material needs to be determined; this cannot be definitively accomplished in situ since the initial mass cannot be measured.

### 3.4. Conclusions

In the present study, RC increased by 33% on Si-supplemented *L. arundinaceum,* suggesting that significant amounts of damage to the plant could occur under Si enrichment. In the context of pest management, Si supplementation could lead to desirable outcomes in terms of reducing pest RGR, but these benefits could be undermined by increased levels of damage to the plant. Nonetheless, Si supplementation can allow plants to tolerate increased levels of herbivory [44,45], and slower herbivore RGR could prolong their exposure to predation and parasitism [46,47]. Indeed, several studies now report that feeding on Si-supplemented plants makes insect herbivores more susceptible to their natural enemies via increased attraction [11,12], compromised immune responses [23,45], or reduced levels of camouflage [48]. Such studies, and earlier reports, have stimulated interest in using Si fertilisation of crops to promote biological control of insect pests [49,50].

In conclusion, while the expectation is that Si supplementation should deter feeding and reduce RC [26,51,52,53], both our results and others [7,32,33], suggest that some generalist insect herbivores may actually cause more feeding damage to Si-supplemented plants. In this study, we show that this may only be possible, however, in plants that do not have high levels of Si defence.

## 4. Materials and Methods

### 4.1. Plant Growth and Experimental Design

Seeds of purple false brome (*Brachypodium distachyon*; Bd21-3) and tall fescue (*Lolium arundinaceum*; cv. INIA Fortuna) were sourced from the French National Institute for Agricultural Research (INRA) and the Margot Forde Germplasm Centre (Palmerston North, New Zealand), respectively. Both plants were cultivated under hydroponic conditions following a modification of the method by Jung et al. [54], subsequently described by Hall et al. [26]. Seeds were initially soaked in water for 2 h to soften the lemma and palea, which were subsequently removed using forceps. Surface sterilisation was performed using a solution containing 0.9% sodium hypochlorite and 0.1% Triton X-100 for 30 min, followed by multiple rinses with sterile water. Sterilised seeds were placed into perlite irrigated with half-strength nutrient solution. After cold stratification at 4 °C for three days, seedlings were grown for 14 days to ensure uniform development before transfer to hydroponic culture, with three seedlings allocated per cup. The hydroponic system comprised two nested disposable cups fitted with a custom-cut foam disc containing three slots to support the plants See Figure S1 in [26]. Each cup was filled with approximately 330 mL of full-strength nutrient solution prepared according to Hall et al. [26].

**Figure 4:**
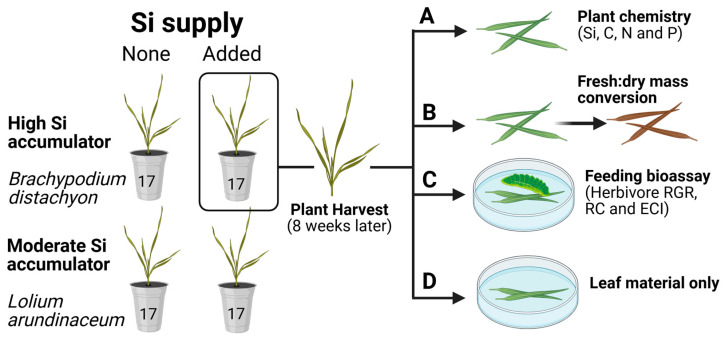
Schematic of experimental design and use of plant material for (**A**) chemistry, (**B**) fresh:dry mass conversion, (**C**) feeding assays, and (**D**) Petri dishes with leaf material only. The latter (**D**) was used to account for water loss during the 72 h feeding trial [26]. Replication: N = 17.

In total, 34 cups for each plant species were used and randomly assigned for Si inclusion (+Si) (N = 17) or maintained as controls without silicon supplementation (−Si) (N = 17) (Figure 4). Si treatments were applied as described by [26] by supplementing the nutrient solution with liquid potassium silicate (K_2_SiO_3_; Agsil32; PQ Australia, Adelaide, Australia) at a concentration equivalent to 2 mM SiO_2_, with the pH adjusted to 5.5 using HCl to minimise silicate polymerisation [55]. Control treatments (-Si) received KCl to equalise potassium and chloride inputs relative to the Si^+^ treatment, and the pH was similarly adjusted to 5.5 with HCl. Experimental cups were randomly arranged within growth chambers, and nutrient solutions were renewed weekly.

The experiment was conducted under natural light conditions. Air temperature was regulated at 24/18 °C (day/night) with a 14 h light:10 h dark photoperiod, and relative humidity was maintained at 60% (±6%). After six weeks, plants were removed from the cups, weighed, and leaf material for each plant was divided for chemical analysis or insect feeding assays (Figure 4).

### 4.2. Plant Elemental Analysis

Elemental analysis was conducted for every plant using leaf tissue designated for this purpose (Figure 4A). Foliar silicon content was quantified using ~100 mg of finely milled leaf tissue loaded into sample cups and analysed by energy-dispersive X-ray fluorescence (Epsilon 3x, Malvern Panalytical, Worcestershire, UK). Measurements followed procedures and calibration against certified reference standards outlined by Reidinger et al. [56]. For elemental chemistry, we determined leaf nitrogen (N) and carbon (C) concentrations via combustion analysis using a FLASH EA 1112 CHN analyser (Thermo Fisher Scientific, Waltham, MA, USA) as previously described [57].

### 4.3. Insect Feeding Assays

To evaluate the effects of silicon supplementation on *Helicoverpa armigera* larval performance, feeding efficiency assays were conducted following methods adapted from Slansky [58] and described by Massey et al. [7,8] and Hall et al. [26]. Individual third-instar larvae (supplied by CSIRO Agriculture & Food), reared on an artificial diet, were starved for 24 h and weighed prior to being transferred to closed Petri dishes containing a pre-weighed portion of fresh leaf tissue (Figure 4C). Larvae were maintained at 24 °C and allowed to feed for 72 h, after which they were starved for a further 24 h to permit frass evacuation before being reweighed. The remaining leaf material in the Petri dish (Figure 4C) was oven-dried at 40 °C and weighed. Water content values, obtained from leaf samples collected from the same plants, were used to convert the initial fresh mass of the grass to dry mass (Figure 4B) [7]. Petri dishes containing cut leaf segments alone were used to account for natural water loss from leaf tissues during the assay (Figure 4D) [26].

Three herbivore performance parameters were quantified: Relative Growth Rate (RGR), Relative Consumption (RC), and Efficiency of Conversion of Ingested Food (ECI).

RGR, defined as body mass increase relative to initial body mass, was calculated as:

$$RGR=\;\frac{\ln\left(W2\right)-\ln\;\left(W1\right)}{t2-t1}$$

where *W*1 is the initial body mass, *W*2 is the final body mass, and *t*2 − *t*1 is the feeding duration (days). RC estimates the mass of food ingested over 72 h relative to initial body mass (mg fresh mass) was calculated as:

$$RC=\;\frac{\Delta \;Massfood\;\left(mg\right)}{Mean\;mass\;body\;\left(mg\;fresh\right)}$$

ECI represents the proportion of consumed food converted into body biomass, and was calculated as the increase in larval fresh body mass (mg) divided by the dry mass of food ingested (mg), multiplied by 100:

$$ECI\;\left(\%\right)=\;\frac{Mass\;gained}{Food\;ingested\;}\times \;100$$

### 4.4. Statistical Analysis

The impacts of Si supplementation on plant and insect response variables (see Table S1) were analysed using a two-way analysis of variance (ANOVA) with plant species, Si supplementation, and their interaction included as fixed factors. Because highly significant differences were observed among plant species for all variables except relative consumption (RC), additional one-way ANOVAs were conducted for each species separately, with Si supplementation as the sole fixed factor. Following correction for water loss (Section 4.3), one RC value and two Efficiency of Conversion of Ingested Food (ECI) estimates fell outside biologically plausible ranges and were excluded from further analysis. Assumptions of homoscedasticity and normality were assessed by visual inspection of residual and quantile–quantile plots. Pearson’s correlation analyses were used to examine relationships between plant elemental composition and insect performance metrics. All analyses were performed in Genstat v. 24 (VSN International Ltd., Hemel Hempstead, UK).

## Data Availability

The data presented in this study are available on request from the corresponding author. The data are not publicly available due to privacy restrictions.

## Supplementary Materials

The following supporting information can be downloaded at https://www.mdpi.com/article/10.3390/plants15091380/s1, Table S1. Results of ANOVA tests for plant and insect responses to Si supplementation comparing grass species (Two-way ANOVA) and examined for each grass species individually (One-way ANOVA). Results shown in bold where *p* < 0.05.
