# Supplementary material for: Plant Silicon Defences Suppress Herbivore Growth but Trigger Compensatory Feeding in a Moderate-Accumulating Grass
Source: Plants (Basel). 2026 Apr 30;15(9):1380. doi: 10.3390/plants15091380 (PMC13165232; doi:10.3390/plants15091380)
Supplement: Supplementary file 1 [file plants-15-01380-s001.zip › plants-4218202-supplementary.pdf]

Table S1. Results of ANOVA tests for plant and insect responses to Si supplementation comparing grass species (Two-way ANOVA) and examined for each grass species individually (One-way ANOVA). Results shown **in bold** where $P < 0.05$.

**Plant / insect response**	**Corresponding Figure**	**Two-way ANOVA**						**One-way ANOVAs**			
								Lolium		*Brachypodium*	
		Plant species		Si		Plant species × Si		Si		Si	
		$F_{1,64}$	*P*	$F_{1,64}$	*P*	$F_{1,64}$	*P*	$F_{1,32}$	*P*	$F_{1,32}$	*P*
Si%	2A	**226.47**	**<0.001**	**794.42**	**<0.001**	**134.97**	**<0.001**	**215.80**	**<0.001**	**580.74**	**<0.001**
C%	2B	**253.98**	**<0.001**	**49.04**	**<0.001**	**36.00**	**<0.001**	0.40	0.533	**115.51**	**<0.001**
N%	2C	**144.25**	**<0.001**	**12.05**	**<0.001**	0.23	0.631	**6.34**	**0.017**	**5.82**	**0.022**
P%	2D	**368.34**	**<0.001**	**23.57**	**<0.001**	1.15	0.287	**11.59**	**0.002**	**14.78**	**<0.001**
C:N	2E	**172.72**	**<0.001**	**4.35**	**0.041**	0.43	0.515	4.10	0.051	0.94	0.338
C:P	2F	**406.62**	**<0.001**	**9.27**	**0.003**	0.15	0.698	**10.75**	**0.003**	3.53	0.070
Si:C	2G	**167.45**	**<0.001**	**708.33**	**<0.001**	**108.65**	**<0.001**	**202.53**	**<0.001**	**507.01**	**<0.001**
Si:N	2H	**242.19**	**<0.001**	**434.94**	**<0.001**	**146.83**	**<0.001**	**129.57**	**<0.001**	**318.75**	**<0.001**
Si:P	2I	**329.42**	**<0.001**	**501.80**	**<0.001**	**206.99**	**<0.001**	**218.13**	**<0.001**	**365.22**	**<0.001**
RGR	3A	**62.42**	**<0.001**	**29.84**	**<0.001**	0.03	0.873	**16.06**	**<0.001**	**13.84**	**<0.001**
RC[a]	3B	3.68	0.06	**7.41**	**0.008**	2.20	0.143	**7.20**	**0.012**	0.98	0.329
ECI[b]	3C	**66.26**	**<0.001**	**26.52**	**<0.001**	0.10	0.748	**12.12**	**0.002**	**15.25**	**<0.001**

Following correction for water loss, one RC and two ECI estimates fell outside biologically feasible ranges and were therefore excluded from analyses. [a]$F_{1,63}$ (two-way ANOVA) and $F_{1,32}$ (one-way ANOVA; *Lolium*); [b]$F_{1,62}$ (two-way ANOVA) and $F_{1,32}$ (both one-way ANOVAs).
